# Ethnic differences in prevalence and behaviors of smoking and their association with chronic obstructive pulmonary disease among the elderly in rural southwest China: A cross-sectional study

**DOI:** 10.18332/tid/209144

**Published:** 2025-10-22

**Authors:** Guo-hui Li, Gui-yi Wang, Lan Liu, Yi Zhao, Xia Wu, Allison R. Golden, Le Cai

**Affiliations:** 1 NHC Key Laboratory of Drug Addiction Medicine, Kunming Medical University, Kunming, China; 2 School of Public Health, Kunming Medical University, Kunming, China; 3 Endocrinology Department, The First Affiliated Hospital of Kunming Medical University, Kunming, China; 4 Department of Public Health Management, The Second Affiliated Hospital of Kunming Medical University, Kunming, China

**Keywords:** smoking, behaviors, chronic obstructive pulmonary disease, ethnicity, China

## Abstract

**INTRODUCTION:**

This study examines how prevalence and behaviors of smoking differ by ethnicity and their associations with chronic obstructive pulmonary disease (COPD) among elderly people of four ethnicities in rural southwest China.

**METHODS:**

A cross-sectional survey of 5642 adults aged ≥60 years was conducted in rural southwest China. Data on the demographics, smoking habits, and post-bronchodilator spirometry were collected.

**RESULTS:**

Among the participants, the prevalence of current smoking (48.8% vs 0.8%) and COPD (12.7% vs 4.5%) was significantly higher in males compared to females (p<0.01). Filtered cigarettes were the most popular form of tobacco used, comprising 76.6% of tobacco consumed. Bai ethnic minority participants had the highest prevalence of current smoking and COPD, and the highest number of cigarettes smoked per day compared to the other three studied ethnicities (p<0.01). Ha Ni ethnic minority participants had the lowest rate of smoking cessation (8.7%) and the highest rate of smoking in public places (66.8%) (p<0.01). The results of multivariable logistic regression indicated that current smokers were more likely to suffer from COPD across all four studied ethnicities (p<0.05). Further, the association of current smoking with COPD in Bai ethnicity elderly participants was stronger compared to other ethnic groups (p<0.01).

**CONCLUSIONS:**

The present study shows that ethnicity plays a significant role in influencing both the prevalence and behaviors related to smoking among elderly people in rural southwest China. Future efforts to prevent and reduce tobacco use in rural China should consider ethnicity, as culturally tailored tobacco control strategies could help prevent and manage the COPD epidemic.

## INTRODUCTION

Smoking causes a wide variety of diseases, chief among them cancer, chronic respiratory disease, and cardiovascular disease (CVD)^1^, and is a major public health challenge worldwide. In 2020, 118 million people smoked globally, resulting in 7 million deaths, or about one-seventh of all deaths^2^. China is the world’s largest producer and consumer of tobacco, accounting for more than one-third of the world’s tobacco consumption in 2019, and almost all tobacco in China is consumed by men^3^. Although smoking prevalence among those aged ≥ 15 years in China declined by 1.7% from 2010 to 2018^4^, this rate of decline was lower than the global average of 3.3%^5^. Prior research has established that smoking is especially harmful to the elderly, causing decreased physical performance and increasing their risk of respiratory diseases^6^. In rural regions of China, the rate of smoking among the elderly greatly exceeds the rate in urban areas (28.3% vs 23.0%)^4^.

Previous studies have explored racial and ethnic disparities in the prevalence of smoking worldwide and have indicated that there are important and persistent ethnic differences in smoking rates among racial and ethnic groups due to differing smoking patterns^7^. While the majority of the Chinese population is of Han ethnicity, China is a multi-ethnic country with 56 ethnic groups. Several Chinese studies have explored the attempt to ban smoking in ethnic minority communities^8^, but the smoking rate among ethnic minority elderly people continues to exceed the rate observed among the Han population in most cases^9^. However, the literature remains sparse, especially with regard to ethnic minority elderly people in rural regions.

Chronic obstructive pulmonary disease (COPD) is a chronic airway inflammation condition characterized by airflow restriction and chronic respiratory symptoms^10^. COPD is increasingly identified as the leading cause of death worldwide. Future morbidity and mortality are expected to increase further due to the aging population, urbanization, and increased environmental pollution^11^. At present, the mortality rate of COPD in China is higher than the global average, and the prevalence rate is increasing, with the fastest increase observed in the elderly^12^. It is well established that smoking is a major risk factor for COPD, and that a variety of harmful substances in tobacco increase airway resistance and cause obstructive injury^13,14^. In addition, elderly smokers may experience a cumulative impact due to continued smoking and are at a higher risk of COPD than non-smokers^15^. However, the association of smoking and COPD among ethnic minority older adults in rural China has not been well investigated thus far.

Yunnan Province is located in southwest China and has a population of 46.9 million people. More than 33% of Yunnan comprises ethnic minorities, making it the province with the largest ethnic minority population in China. Of these, 7 million are elderly. Previous research has uncovered that smoking is highly prevalent in this region of China^16^. However, there are limited data on the prevalence and patterns of smoking among the ethnic minority elderly population in rural China, as well as how prevalence and behaviors of smoking relate to COPD and vary by ethnicity. Thus, this study aimed to examine ethnic differences in prevalence and behaviors of smoking among the Han majority and three unique ethnic minority groups (Dai, Ha Ni, and Bai), and to investigate the relationship between smoking and COPD among southwest China’s rural elderly adult population aged ≥60 years.

## METHODS

### Study area and population

This study used a community-based cross-sectional health interview and survey across three regions of Yunnan Province from 2021 to 2022. One minority autonomous prefecture or county was randomly selected from each of the three habitated environments of the 15 unique minorities in Yunnan Province: dam area, river valley, and mountainous region. A consistent three-stage stratified random sampling selection process was employed to select elderly individuals aged ≥60 years of ethnic minority and Han descent. This sampling method has been previously described in detail^17^. Inclusion criteria were: 1) participants aged ≥60 years; and 2) participants residing in the selected villages for ≥5 years and willing to participate in the study. Exclusion criteria were: 1) participants aged <60 years; 2) those with cognitive dysfunction or inability to communicate with the interviewers; and 3) those who refused to provide informed consent to participate in the study. To facilitate statistical comparison across ethnic groups and ensure adequate statistical power, we adopted an approximately equal sampling strategy across the four ethnic groups. Although this does not reflect the actual ethnic composition in the general population of Yunnan, it allows for better within-group analyses and comparison of smoking behaviors and COPD outcomes.

*Sample size calculation*

The sample size for each selected village was estimated using the standard formula for cross-sectional studies:


$$n=z_{1-a/2}^{2}\frac{\left(1-p\right)p}{\delta^{2}}\times deff$$

where *p* represents the estimated prevalence of COPD among older adults in China^18^, δ is the allowed margin of error (defined as half of the estimated prevalence), and *deff* denotes the design effect (*deff*=2). This sample size was considered adequate to ensure a statistical power of at least 80% for estimating the expected prevalence of COPD.

### Data collection and measurement

After giving informed consent, each participant was interviewed face-to-face by trained interviewers using a pre-tested and structured questionnaire. The questionnaire included age, gender, ethnicity, annual household income, level of education, and self-reported smoking habits. Anthropometric measurements, including height and weight, and post-bronchodilator pulmonary function tests were also recorded.

Height and weight were measured with standardized equipment using the WHO STEPS procedure^19^. Body mass index (BMI) was calculated as weight (kg) divided by height squared (m^2^).

Physicians used a portable data logging spirometer (CHEST HI-101) to perform spirometry in all participants and measure post-bronchodilator pulmonary function tests, according to American Thoracic Society and European Respiratory Society (ATS/ERS) guidelines^20^. Participants began the test in a sitting position after a 5-minute rest, with exhalation before each test. A short-acting bronchodilator (salbutamol 400 μg) was administered via a metered-dose inhaler with a spacer. Spirometry was performed before and 15 minutes after bronchodilator administration. Measurements taken included forced expiratory volume in the first second (FEV1) and forced vital capacity (FVC), and the ratio was calculated (FEV1/FVC). FEV1/FVC <0.7 was the cutoff used to perform post-bronchodilator spirometry. All participants had at least three measurements taken, and the highest measurement was used in our analysis.

### Definitions

According to the Global Initiative for Chronic Obstructive Lung Disease (GOLD), COPD is defined as FEV1/FVC <0.7 after inhalation of bronchodilators, characterized by persistent airflow limitation^21^. Current smokers are defined as those who smoke continuously or cumulatively for 6 months or more in their lifetime, have smoked more than 100 cigarettes cumulatively, or currently smoke any kind of tobacco product on a daily basis^22^. Illiteracy was defined as the inability to either read with understanding or to write a simple sentence about everyday life. Annual household income was defined as low (<965 US$) or high (≥965 US$), with the median value as the cut-off point.

### Statistical analysis

EpiData 3.1 software (EpiData Association, Odense, Denmark)^23^ was used for double data entry, and SPSS 22.0 software (IBM Corp, Armonk, NY, USA)^24^ was used for data analysis. Values of FEV1, FVC, FEV1/FVC, and BMI were expressed as mean and standard deviation when these parameters were normally distributed, otherwise they were expressed as median and interquartile range (IQR). Categorical variables were described as frequencies and percentages. One-way ANOVA was used to analyze continuous measures, while the chi-squared test was used to compare categorical variables. Multivariable logistic regression analysis was used to analyze the association between current smokers and the prevalence of COPD adjusted by sex, age, education level, annual household income, and BMI. The factors associated with COPD were selected based on biological plausibility and were initially evaluated using univariable logistic regression analysis. Variables with p<0.05 in the univariable analysis were considered as candidate variables for inclusion in the multivariable logistic regression model. Associations were expressed as odds ratios and 95% confidence intervals (CIs). All statistical significance decisions were based on two-tailed p<0.05.

## RESULTS

Individuals from the Han majority ethnicity and three ethnic minority groups (Dai, Ha Ni, and Bai) were selected, with a total of 5800 individuals aged ≥60 years invited to participate in the study. Of these, 5642 consented, for an overall response rate of 97.28%.

Table 1 shows the demographic characteristics of the participants. Of the 5642 participants, there were 2718 (48.2%) males and 2924 (51.8%) females. The Han, Dai, Ha Ni, and Bai ethnicities accounted for 25.0%, 25.0%, 24.8%, and 25.1% of participants, respectively. There was no significant difference in the proportion of males (p>0.05). Han ethnicity participants had the highest level of education, whereas Ha Ni ethnic minority participants had the lowest. Bai ethnic minority participants had the highest level of annual household income and mean BMI, while Ha Ni had the lowest (p<0.01).

**Table 1:** Demographic characteristics of the study population, a cross-sectional study in rural Yunnan Province, China, 2021–2022 (N=5642)

*Characteristics*	*Han ethnic majority n (%)*	*Dai ethnic minority n (%)*	*Ha Ni ethnic minority n (%)*	*Bai ethnic minority n (%)*	*All n (%)*
**Sex^a^**					
Male	686 (48.5)	690 (49.0)	673 (48.0)	669 (47.2)	2718 (48.2)
Female	727 (51.5)	719 (51.0)	729 (52.0)	749 (52.8)	2924 (51.8)
**Age^a^** (years)					
60–64	227 (16.1)	367 (26.0)	409 (29.2)	223 (15.7)	1226 (21.7)
65–69	465 (32.9)	446 (31.7)	420 (30.0)	481 (33.9)	1812 (32.1)
70–74	367 (26.0)	328 (23.3)	295 (21.0)	335 (23.6)	1325 (23.5)
≥75	354 (25.1)	268 (19.0)	278 (19.8)	379 (26.7)	1279 (22.7)
**Education level^a^**					
Illiterate	558 (39.5)**	729 (51.7)**	864 (61.6)**	581 (41.0)**	2732 (48.4)**
Primary school (grades 1–6) or higher	855 (60.5)	680 (48.3)	538 (38.4)	837 (59.0)	2910 (51.6)
**Annual household income^a^** (US$)					
Low (<965)	742 (52.5)**	646 (45.8)**	826 (58.9)**	607 (42.8)**	2821 (50.0)**
High (≥965)	671 (47.5)	763 (54.2)	576 (41.1)	811 (57.2)	2821 (50.0)
**Spirometry^b^,** mean ± SD					
FEV1 (L)	1.69 ± 0.52	1.72 ± 0.53	1.53 ± 0.52*	1.70 ± 0.51	1.66 ± 0.53**
FVC (L)	2.10 ± 0.61*	2.11 ± 0.62*	1.92 ± 0.58*	2.19 ± 0.61*	2.08 ± 0.61**
FEV1/FVC (%)	80 ± 8*	82 ± 10*	79 ± 9*	77 ± 9*	79 ± 9**
**Mean BMI^b^** (kg/m^2^)	22.84 ± 3.65*	21.76 ± 3.66*	21.52 ± 3.24*	23.73 ± 3.43*	22.46 ± 3.61**

BMI: body mass index. FEV1: forced expiratory volume in one second. FVC: forced vital capacity.

a Differences in sex, age, level of education, and level of annual household income among four ethnic groups were analyzed by chi-square test.

b Differences in FEV1, FVC, FEV1/FVC and mean BMI among four ethnic groups were analyzed by independent two-sample test.

* p<0.05,

** p<0.01.

Table 2 presents the prevalence of smoking behaviors among current smokers in the study population by ethnicity. The overall prevalence rate of current smoking was 23.9%, and males had significantly higher prevalence of current smoking than females (48.8% vs 0.8%, p<0.01). The prevalence of current smoking and behaviors of smoking differed significantly by ethnicity. Filtered cigarettes were the most popular form of tobacco consumption, comprising 76.6% of all tobacco consumed among the four studied ethnicities, followed by the use of a hookah (waterpipe) in Han (18.3%) and Dai populations (34.0%), tobacco pipes in the Ha Ni population (27.2%), and hand-rolled cigarettes in the Bai population (7.6%). Bai ethnicity participants smoked the highest number of cigarettes per day and also had the highest prevalence of current smoking, compared to the other three studied ethnicities (p<0.01). Ha Ni ethnicity participants had the lowest rate of smoking cessation (8.7%) within one year and the highest rate of smoking in public places (66.8%) in the seven days prior to the study (p<0.01).

**Table 2:** Prevalence of current smoking and smoking behaviors among current smokers by ethnicity, a cross-sectional study in rural Yunnan Province, China, 2021–2022 (N=5642)

*Variables*	*Han ethnic majority n (%)*	*Dai ethnic minority n (%)*	*Ha Ni ethnic minority n (%)*	*Bai ethnic minority n (%)*	*All n (%)*
**Use of various forms of tobacco^a^**					
Filtered cigarettes	247 (83.7)**	182(69.5)**	188 (56.3)**	417 (91.0)**	1034 (76.6)**
Unfiltered cigarettes	6 (2.0)	13 (5.0)**	9 (2.7)	6 (1.3)**	34 (2.5)*
Hookah	54 (18.3)**	89 (34.0)**	90 (26.9)**	13 (2.8)**	246 (18.2)**
Pipe	9 (3.1)	3 (1.1)	91 (27.2)**	13 (2.8)	116 (8.6)**
Hand-rolled cigarettes	8 (2.7)**	3 (1.1)**	25 (7.5)**	35 (7.6)**	71 (5.3)**
Chewing tobacco	0 (0.0)	0 (0.0)	0 (0.0)	0 (0.0)	0 (0.0)
Cigarettes per day^b^, mean ± SD	15.18 ± 8.78**	12.15 ± 7.77**	12.51 ± 8.83**	17.49 ± 9.23**	15.02 ± 9.05**
**Previous quit attempts^a^**					
No attempt	204 (69.2)	203 (77.5)	305 (91.3)**	340 (74.2)	1052 (78.0)**
At least one attempt in previous 12 months	91 (30.8)	59 (22.5)	29 (8.7)	118 (25.8)	297 (22.0)
**Location of smoking in the prior seven days^a^**					
Public spaces (village, schools, hospitals, etc.)	131 (44.4) **	103 (39.3) **	223 (66.8) **	295 (64.4) **	752 (55.7) **
Home	164 (55.6)	159 (60.7)	111 (33.2)	163 (35.6)	597 (44.3)
**Current smokers^a^**					
Male	290 (42.3) **	256 (37.1) **	330 (49.0) **	451 (67.4) **	1327 (48.8) **
Female	5 (0.7)	6 (0.8)	4 (0.5)	7 (0.9)	22 (0.8)
All	295 (20.9)**	262 (18.6)**	334 (23.8)**	458 (32.3)**	1349 (23.9)**

a Differences in use of various forms of tobacco, previous quit attempts, location of smoking in the prior seven days, and current smokers among four ethnic groups were analyzed by chi-square test.

b Differences in mean number of cigarettes per day among four ethnic groups were analyzed by independent two-sample test.

* p<0.05,

** p<0.01.

**Figure 1:**
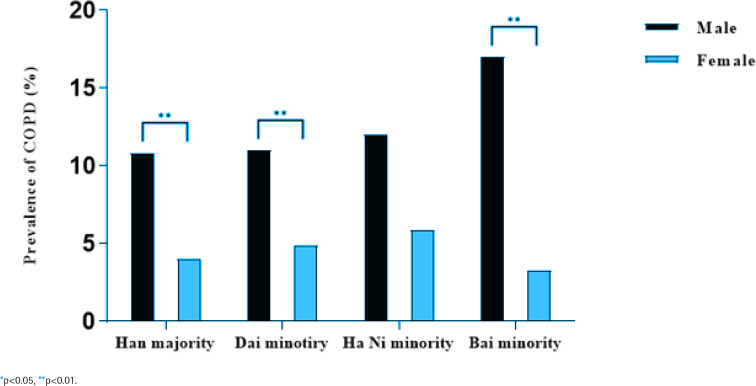
Prevalence of COPD by sex and ethnicity, a cross-sectional study in rural Yunnan Province, China, 2021–2022 (N=5642)

**Figure 2:**
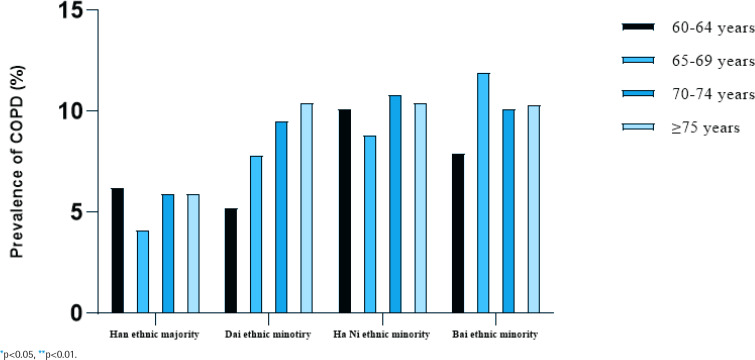
Prevalence of COPD by age group and ethnicity, a cross-sectional study in rural Yunnan Province, China, 2021–2022 (N=5642)

Table 3, and Figures 1 and 2, indicate the prevalence of COPD by ethnicity, sex, and age. The overall prevalence of COPD in the study population was 8.5% (12.7% for males and 4.5% for females), and the prevalence of COPD increased with age (p<0.01). In all four studied ethnicities, males had a higher prevalence of COPD than females (p<0.01). Bai ethnic minority males had the highest prevalence of COPD (p<0.01), but there was no significant difference in prevalence of COPD among the four studied female populations (p>0.05).

**Table 3:** Prevalence of chronic obstructive pulmonary disease by ethnicity, sex, and age, a cross-sectional study in rural Yunnan Province, China, 2021–2022 (N=5642)

*Variables*	*Han ethnic majority n (%)*	*Dai ethnic minority n (%)*	*Ha Ni ethnic minority n (%)*	*Bai ethnic minority n (%)*	*All n (%)*
**Sex^a^**					
Male	74 (10.8)**	76 (11.0)**	81 (12.0)	114 (17.0)**	345 (12.7)**
Female	29 (4.0)	35 (4.9)	43 (5.9)	25 (3.3)	132 (4.5)
**Age^a^** (years)					
60–64	14 (6.2)	15 (4.1)	24 (5.9)	15 (5.9)	68 (5.5)
65–69	24 (5.2)	35 (7.8)	40 (9.5)	50 (10.4)	149 (8.2)
70–74	37 (10.1)	29 (8.8)	32 (10.8)	35 (10.4)	133 (10.0)
≥75	28 (7.9)	32 (11.9)	28 (10.1)	39 (10.3)	127 (10.1)**
All	103 (7.3)	111 (7.9)	124 (8.8)	139 (9.8)*	477 (8.5)

a Differences in proportion of male and age group among four ethnic group were analyzed by chi-squared test.

* p<0.05,

** p<0.01

Table 4 displays the results of the multivariable logistic regression analysis of the prevalence of COPD. In all four studied ethnicities, males had a higher probability of suffering from COPD than females (p<0.01). Older adults with a lower level of education and annual household income were more likely to suffer from COPD (p<0.01). Current smokers had a greater probability of suffering from COPD (p<0.05), with the association between current smoking and COPD appearing more pronounced in the Bai population compared to other ethnic groups (p<0.01).

**Table 4:** Multivariable logistic regression for prevalence of chronic obstructive pulmonary disease, a cross-sectional study in rural Yunnan Province, China, 2021–2022 (N=5642)

*Variables*	*COPD (Ref: No)*			
	*Han ethnic majority OR (95% CI)*	*Dai ethnic minority OR (95% CI)*	*Ha Ni ethnic minority OR (95% CI)*	*Bai ethnic minority OR (95% CI)*
**Sex**	0.38**(0.24–0.60)	0.40**(0.26–0.62)	0.38**(0.24–0.61)	0.16**(0.01–0.26)
(Ref: Male)				
**Age (years)**	1.22 (1.00–1.49)	1.35**(1.12–1.64)	1.15 (0.97–1.38)	1.13 (0.95–1.34)
(per 5-year increase)				
**Education level**	0.75**(0.64–0.87)	0.83**(0.61–0.92)	0.80**(0.63–0.91)	0.63**(0.48–0.82)
(Ref: Illiterate)				
**Annual household income**	0.76**(0.66–0.87)	0.84**(0.69–0.93)	0.73**(0.60–0.88)	0.67**(0.53–0.84)
(Ref: Low)				
**BMI (kg/m^2^)**	1.00 (0.94–1.06)	0.89**(0.84–0.95)	0.83**(0.77–0.89)	0.96 (0.90–1.01)
(per 1 unit increase)				
**Current smoker**	2.06**(1.42–2.99)	1.67*(1.12–2.50)	2.58**(1.72–3.86)	3.32**(1.94–5.73)
(Ref: No)				

* p<0.05,

** p<0.01.

## DISCUSSION

The findings of the present study indicate significant ethnic differences in the prevalence of current smoking and smoking behaviors. Further, it revealed that current smokers had a greater probability of suffering from COPD, though the intensity of the association of current smoking with COPD varied by ethnicity.

The overall prevalence of current smoking in the four studied ethnic groups in the present study was higher than recorded in other regions of China^25,26^ as well as other Asian countries^27^, indicating smoking is highly prevalent among the elderly in rural areas of southwest China. Furthermore, among the four ethnic groups investigated, males smoked more frequently than females, a gender pattern that has held consistently historically and has been demonstrated in many previous studies^2,28^. The present study also revealed significant ethnic differences in the prevalence of current smoking, with Bai ethnic minority participants having the highest prevalence of current smoking and smoking the highest number of cigarettes per day. There were ethnic differences in the prevalence of smoking, which have also been found in previous studies^9^. In this way, our results suggest that smoking cessation programs are urgently needed in rural areas of southwest China, particularly programs targeting men and those of the Bai ethnic minority.

The present study also uncovered that filtered cigarettes were the most popular form of tobacco consumption in all four studied ethnicities, a finding consistent with previous research in rural southwest China^9^. Additionally, hookah usage was also popular in the Dai ethnic minority population, and tobacco pipe use was common amongst those of the Ha Ni ethnic minority. These findings illustrate that cultural forces may play an important role in shaping smoking habits.

Our study also found that Ha Ni ethnic minority elderly participants had the lowest rate of smoking cessation (8.7%) within one year of the study, and the highest rate of smoking (66.8%) in public places in the seven days prior to the study. This possibly results from the fact that Ha Ni elderly participants had the lowest level of education among the four studied ethnic groups, as previous research indicated an inverse association between education and both willingness to quit smoking and likelihood of smoking in public places^29,30^. Our findings thereby suggest programs to raise awareness about the harms of tobacco use to improve willingness to quit smoking should focus in particular on the Ha Ni ethnicity elderly in the surveyed communities.

The prevalence of COPD was markedly higher in men than in women, and it increased with age, which is consistent with results recorded worldwide^31^. Furthermore, there were significant ethnic differences in the prevalence of COPD among the four studied ethnicities, with Bai ethnicity elderly participants recording the highest prevalence of COPD. This result is consistent with previous research in China^32^. After controlling for potential confounders, including sex, age, education level, income, and BMI, the results uncovered that current smokers had a higher risk of COPD across all four ethnic groups. Smoking is an important contributor to COPD, as has been demonstrated in many previous studies^33,34^. We additionally found the intensity of the association of current smoking with COPD varied by ethnicity, a pattern that has been observed in other studies as well^35^. In our study, the association of current smoking with COPD in Bai ethnicity elderly participants was stronger than in other ethnic groups. The possible explanation for this trend is the fact that Bai ethnic minority participants had the highest current smoking rate and smoked the highest number of cigarettes per day. Previous research has also shown that the Bai ethnic minority tends to have higher smoking rates compared to other ethnic groups^36^. Our findings emphasize that public health strategies for tobacco control and COPD prevention must take ethnicity factors into account and adopt culturally appropriate interventions.

### Limitations

The following limitations of this study should be noted. First, the self-reported prevalence of smoking may be influenced by recall bias, potentially leading to an underestimation of the true smoking prevalence within the population. Second, the cross-sectional design of this study limits the ability to determine causal relationships. Third, the generalizability of the present findings is constrained by the utilization of a random sampling technique as well as the fact that it included only four ethnic groups. Fourth, although we adjusted for multiple covariates in our models, the possibility of residual confounding due to unmeasured or inadequately measured variables cannot be ruled out. Fifth, the approximately equal distribution of participants across the four ethnic groups, rather than reflecting their actual population proportions, may limit the generalizability of prevalence estimates. Finally, we were unable to obtain data on the whole population of Yunnan Province stratified by sex, age, and ethnicity. Consequently, we could not perform weighted analyses on the data, which may limit the generalizability of the results to all rural regions in Yunnan Province.

## CONCLUSIONS

The present study indicates ethnicity plays an important role in influencing both prevalence and behaviors of smoking among elderly people in rural areas of southwest China. Future interventions to prevent and reduce tobacco consumption in rural China should address ethnicity, as culturally appropriate tobacco control interventions could help prevent and manage the COPD epidemic.

## Data Availability

The data supporting this research are available from the authors on reasonable request.
